# Fertility-sparing surgery in primary peritoneal serous borderline tumor: a case report

**DOI:** 10.3389/fonc.2025.1480730

**Published:** 2025-05-29

**Authors:** Tingting Li, Wei Jiang, Hongjing Wang

**Affiliations:** ^1^ Department of Gynecology and Obstetrics, West China Second University Hospital, Sichuan University, Sichuan, Chengdu, China; ^2^ Key Laboratory of Birth Defects and Related Diseases of Women and Children (Sichuan University), Ministry of Education, Sichuan, Chengdu, China; ^3^ Department of Pathology, West China Second University Hospital, Sichuan University, Sichuan, Chengdu, China

**Keywords:** PPSBT: primary peritoneal serous borderline tumor, FSS: fertility-sparing surgery, SBT: ovarian serous borderline tumors, ovarian lesion, case report

## Abstract

Primary peritoneal serous borderline tumor (PPSBT) is a rare, low-malignant-potential neoplasm arising from the peritoneum, diagnosed only after excluding ovarian involvement. While typically discovered incidentally during surgery, it often presents with infertility or abdominal pain in young women. Due to its favorable prognosis and the desire to preserve fertility, fertility-sparing surgery (FSS) is a critical consideration. We report a case of PPSBT in a reproductive-aged woman who underwent three FSS procedures, demonstrating the feasibility of this approach. Our findings support FSS as a viable option for PPSBT patients after thorough exclusion of ovarian malignancy. This case underscores the importance of comprehensive surgical staging and multidisciplinary evaluation to optimize oncological and reproductive outcomes. Further research is needed to standardize management strategies for this rare condition.

## Introduction

Extra-ovarian peritoneal implants are observed in 30–50% of women with ovarian serous borderline tumors (SBTs) (1). However, similar peritoneal lesions have been reported in cases with no ovarian involvement or only minimal surface involvement. These lesions have been described using various terms, including atypical endosalpingiosis, primary papillary peritoneal neoplasia, serous micropapillomatosis of low malignant potential, and serous papillary borderline tumors of the peritoneum (2). Currently, the most widely accepted terminology is PPSBT (3), which emphasizes its histological resemblance to non-invasive implants of SBTs. PPSBT is a rare, low-malignant-potential neoplasm arising from the peritoneum. It is diagnosed only when ovarian involvement is absent, distinguishing it from secondary peritoneal implants of ovarian SBTs (4). Most reported cases of PPSBT were incidentally detected during laparotomy or laparoscopy (2, 4, 5). The majority of affected women (>80%) are premenopausal, typically between 30 and 40 years of age (2). Common clinical presentations include infertility and abdominal pain. Although standardized treatment guidelines for PPSBT are lacking, surgical debulking of peritoneal lesions remains the primary approach. Given its excellent prognosis and frequent occurrence in young women of reproductive age, FSS should be considered after excluding an ovarian primary tumor. Here, we present a case of PPSBT in a patient who underwent three FSS procedures.

## Case report

**Figure 1:**
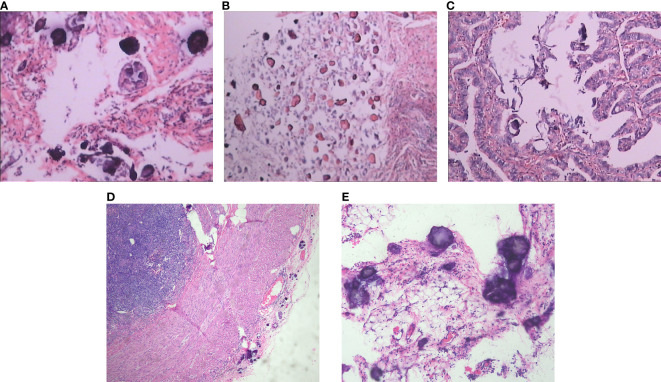
Pathological findings of the specimens obtained from the three surgeries. **(A)** The nodules on the posterior uterine wall (HEx200). **(B)** The tumors on the pelvic region showed Invasive and non-invasive implant foci of borderline serous adenoma with sand formation (HEx200). **(C)** The tumors on the right fallopian tube showed Invasive and non-invasive implant foci of borderline serous adenoma with sand formation (HEx200). **(D)** Appendix demonstrated a large number of sand grains and borderline serous tumors(HEx40). **(E)** Omentum demonstrated a large number of sand grains and borderline serous tumors (HEx100).

The patient was a 33-year-old nulliparous woman presenting with secondary infertility, admitted to West China Second University Hospital, Sichuan University. Hysterosalpingography revealed intrauterine adhesions and bilateral fallopian tube adhesions. The patient denied any personal or family history of malignancy. Her medical history was significant for two failed *in vitro* fertilization-embryo transfer (IVF-ET) cycles due to infertility, both resulting in spontaneous abortions. These procedures necessitated repeated intrauterine manipulations, which subsequently led to the formation of intrauterine and tubal adhesions. Serum tumor marker levels were within normal limits. Transvaginal ultrasound confirmed intrauterine adhesions, while hysterosalpingography demonstrated bilateral tubal occlusion. Given the patient’s strong reproductive desire, she underwent combined hysteroscopic and laparoscopic adhesiolysis as the primary surgical intervention. Laparoscopic exploration revealed multiple disseminated miliary nodules on the posterior uterine wall, anterior rectal wall, bilateral fallopian tubes, and pelvic sidewalls surrounding the ovaries. Surgical management included hysteroscopic adhesiolysis, laparoscopic salpingolysis, and excision of pelvic lesions. Notably, post-adhesiolysis examination showed no macroscopic evidence of ovarian or tubal neoplasia. Intraoperative frozen section analysis of the posterior uterine wall lesions showed serous tumors with extensive psammomatous calcification. Final histopathological examination confirmed non-invasive implants of a serous borderline tumor with psammoma bodies (**Figure 1A**). The primary origin of these lesions remained undetermined. Following multidisciplinary consultation and thorough discussion of the risks, the patient elected for conservative management with close surveillance. The follow-up protocol included regular transvaginal ultrasonographic evaluation of the uterus and adnexa, along with serial tumor marker assessment.

Notably, the second surgery was performed 16 months after the initial procedure. The patient was admitted to Xinan Gynecological Hospital in Chengdu for in IVF-ET. Preoperative ultrasound findings indicated hydrosalpinx, prompting a combined hysteroscopy and laparoscopy. Due to limited exposure of the right fallopian tube, a left salpingectomy and right tubal ligation were performed. During laparoscopy, a well-demarcated, grayish-white nodule (approximately 1 cm in diameter) was observed near the right pelvic sidewall. Additionally, multiple small miliary nodules were identified in the right paracolic gutter and upper abdominal region. Given these findings, the patient’s family was consulted intraoperatively, and a biopsy of the pelvic nodule was performed. Histopathological examination confirmed the presence of both invasive and non-invasive implants of a serous borderline tumor with psammoma bodies.

One month after the second surgery, the patient returned to West China Second University Hospital, Sichuan University, for evaluation of suspected tumor progression. Pathological consultation revealed disseminated pelvic lesions and right fallopian tube involvement, characterized by invasive and non-invasive implants of a serous borderline tumor with psammoma bodies (**Figures 1B, C**). Peritoneal lavage cytology further confirmed a serous borderline tumor, likely of adnexal origin. Intriguingly, laparoscopy showed no macroscopic ovarian tumors. Given these findings, the patient was diagnosed with advanced serous borderline ovarian tumor, prompting cytoreductive surgery with comprehensive staging. After obtaining detailed informed consent, the procedure included: Pelvic lymph node dissection, Para-aortic lymph node sampling, Left salpingectomy, Omentectomy, Appendectomy, Tumor debulking (achieving no visible residual disease). Since prior surgeries had not performed ovarian biopsies, bilateral ovarian biopsies were conducted to assess occult lesions. Histopathology confirmed serous borderline tumor implants with psammoma bodies in the omentum, appendix, and pelvic peritoneum (**Figures 1D, E**).

According to World Health Organization (WHO) criteria, the tumor was classified as borderline malignancy. Due to the absence of ovarian involvement, this case was defined as PPSBT. The patient received four cycles of chemotherapy (cyclophosphamide + cisplatin) and remained disease-free for 8 years post-surgery. However, 6 years after FSS, she underwent bilateral oophorectomy due to encapsulated ovarian effusions, resulting in permanent loss of reproductive function.

## Discussion and conclusions

PPSBT is a rare neoplasm of the peritoneal cavity with potential malignant behavior, hypothesized to arise from remnants of the secondary Müllerian system embedded within or adjacent to the peritoneum (6). Typically, PPSBT is discovered incidentally during surgical procedures. While it predominantly affects women of reproductive age, isolated cases have been reported in postmenopausal individuals (7). Clinically, PPSBT most commonly manifests with two primary symptoms: infertility and abdominal pain. Limited research exists on its risk factors, though current evidence suggests an association with nulliparity and infertility; however, the role of hereditary factors remains unclear (2).

In our case, PPSBT was incidentally discovered during diagnostic laparoscopy for infertility. Laparoscopic examination revealed pelvic peritoneal involvement, manifesting as diffuse adhesions, small nodules, or miliary granularity. Histologically, PPSBT exhibits features resembling non-invasive implants of low malignant potential serous ovarian neoplasms.

The definitive diagnosis was confirmed by pathological examination, which identified epithelial and desmoplastic non-invasive lesions without ovarian involvement. Notably, psammoma bodies and endosalpingiosis were observed in up to 88% of cases (1). As pathological examination remains the gold standard for diagnosing gynecological tumors, the differential diagnosis of PPSBT is critical. Key considerations include: Endometriosis and endosalpingiosis; Benign reactive mesothelial proliferations (e.g., adenomatoid tumor or florid mesothelial hyperplasia); Borderline mesothelial proliferations with overlapping histological features, such as benign or well-differentiated papillary mesothelioma (5, 8). Among neoplastic differentials, primary ovarian papillary serous borderline tumor with peritoneal implants and high-grade primary peritoneal papillary serous carcinoma are the most clinically significant (9). To date, no comparative studies have systematically distinguished PPSBT from primary ovarian serous borderline tumors. Thus, conventional histomorphological analysis combined with microscopic evaluation remains the cornerstone of diagnosis.

Standardized therapeutic guidelines for PPSBT remain undefined. Historically, the recommended treatment involved total abdominal hysterectomy with bilateral salpingo-oophorectomy and omentectomy; however, the clinical benefits of this aggressive approach lack robust evidence. Currently, the primary management for PPSBT consists of surgical debulking of peritoneal lesions. For young women desiring fertility preservation, fertility-sparing surgery (FSS) may be considered after thorough exclusion of an ovarian primary tumor. The role of adjuvant therapy in PPSBT remains controversial. Chemotherapy demonstrates limited efficacy, with only a minority of patients exhibiting a response (10). In a study by Janna et al. (2), adjuvant chemotherapy or radiation was administered in 15.3% of cases, yet no significant prognostic improvement was observed, suggesting that such interventions may be unnecessary. Notably, PPSBT is associated with an excellent prognosis, characterized by a low risk of recurrence or malignant transformation to low-grade serous carcinoma. Long-term survival rates mirror those of women with serous borderline ovarian tumors and non-invasive peritoneal implants, with a 95% survival rate over a mean follow-up period of 6.6 years (11). Furthermore, successful pregnancies following conservative treatment have been documented, highlighting the feasibility of fertility preservation in select cases (4).

Although FSS is a viable treatment option for young patients with PPSBT, its clinical application poses significant challenges in disease management. A key difficulty lies in the initial diagnosis of PPSBT, as these cases are often detected incidentally during laparotomy or laparoscopy, typically in the context of infertility and extensive pelvic adhesions. In this report, we present the case of a female patient with PPSBT who underwent FSS on three separate occasions. Notably, despite repeated interventions, the patient did not achieve a successful pregnancy, a outcome likely influenced by multiple contributing factors.

The preoperative evaluation was constrained by two key factors: (i) the patient’s prior IVF-ET treatment at an external institution obscured clinical suspicion of malignancy, and (ii) the absence of computed tomography(CT)/magnetic resonance imaging(MRI) before both surgical interventions due to the unexpected tumor diagnosis. This lack of cross-disciplinary consultation particularly manifested in three aspects: First, diagnostic monitoring proved inadequate during follow-up. While serial transvaginal ultrasounds and tumor marker assays were performed, advanced imaging surveillance (CT/MRI) was omitted. Second, the assisted reproduction team exclusively addressed hydrosalpinx management prior to the second surgery without comprehensive oncological evaluation. Third, no individualized treatment plan was established through tumor board discussion post-diagnosis, despite the patient’s unique profile of having undergone two failed IVF-ET cycles with consequently limited natural conception potential.

The case highlights the growing imperative for multimodal treatment approaches in fertility-preserving oncology care. A notable opportunity existed during the initial FSS: prophylactic bilateral salpingectomy could have potentially averted subsequent hydrosalpinx-related adhesions while simultaneously reducing cumulative surgical trauma to the pelvic environment. This strategy finds support in Marchette et al.’s (12) findings that while surgical approach (conservative vs. radical) does not affect recurrence rates or fertility outcomes in serous borderline ovarian tumors, repeated surgical interventions demonstrably compromise residual fertility potential.

In the present case, the patient primarily exhibited extraovarian implants, and PPSBT was not initially considered due to the absence of an identifiable primary lesion. This diagnostic oversight is not uncommon and may lead to delayed recognition of PPSBT, ultimately influencing surgical decision-making. Given the patient’s strong desire for fertility preservation and the potential risks associated with repeated surgeries, close postoperative surveillance was implemented following the first FSS. Subsequent pathological analysis from the second FSS suggested an adnexal origin of the tumor. Notably, no distinct lesions were detected in the bilateral ovaries during any of the three FSS procedures, including the third surgery, in which ovarian biopsies also revealed no evidence of tumor involvement. This absence of ovarian pathology may have contributed to the initial misdiagnosis, complicating the differentiation between PPSBT and serous borderline ovarian tumor.

Finally, restaging surgery was not performed in a timely manner. Although no gross lesions were detected in the bilateral ovaries, restaging—along with the identification of the primary lesion based on the initial FSS pathology—remained a critical step. Notably, preoperative positron emission tomography (PET) imaging was not conducted for this patient, which could have aided in surgical staging. Due to the unclear origin of the primary lesion, a definitive diagnosis could not be established. Moreover, complete surgical staging was omitted, and not all potential peritoneal lesions were resected. Importantly, however, the patient remained relapse-free after undergoing comprehensive surgical staging.

The findings of this study highlight the importance of considering FSS for young patients with PPSBT. Future research should prioritize multidisciplinary collaboration to optimize diagnostic and therapeutic strategies. Additionally, this study emphasizes that comprehensive surgical staging plays a pivotal role in fertility preservation and management for this patient population.

## Data Availability

The original contributions presented in the study are included in the article/**Supplementary Material**. Further inquiries can be directed to the corresponding author.
