# Supplementary figures and images for: Fertility-sparing surgery in primary peritoneal serous borderline tumor: a case report
Source: Front Oncol. 2025 May 29;15:1480730. doi: 10.3389/fonc.2025.1480730 (PMC12158697; doi:10.3389/fonc.2025.1480730)

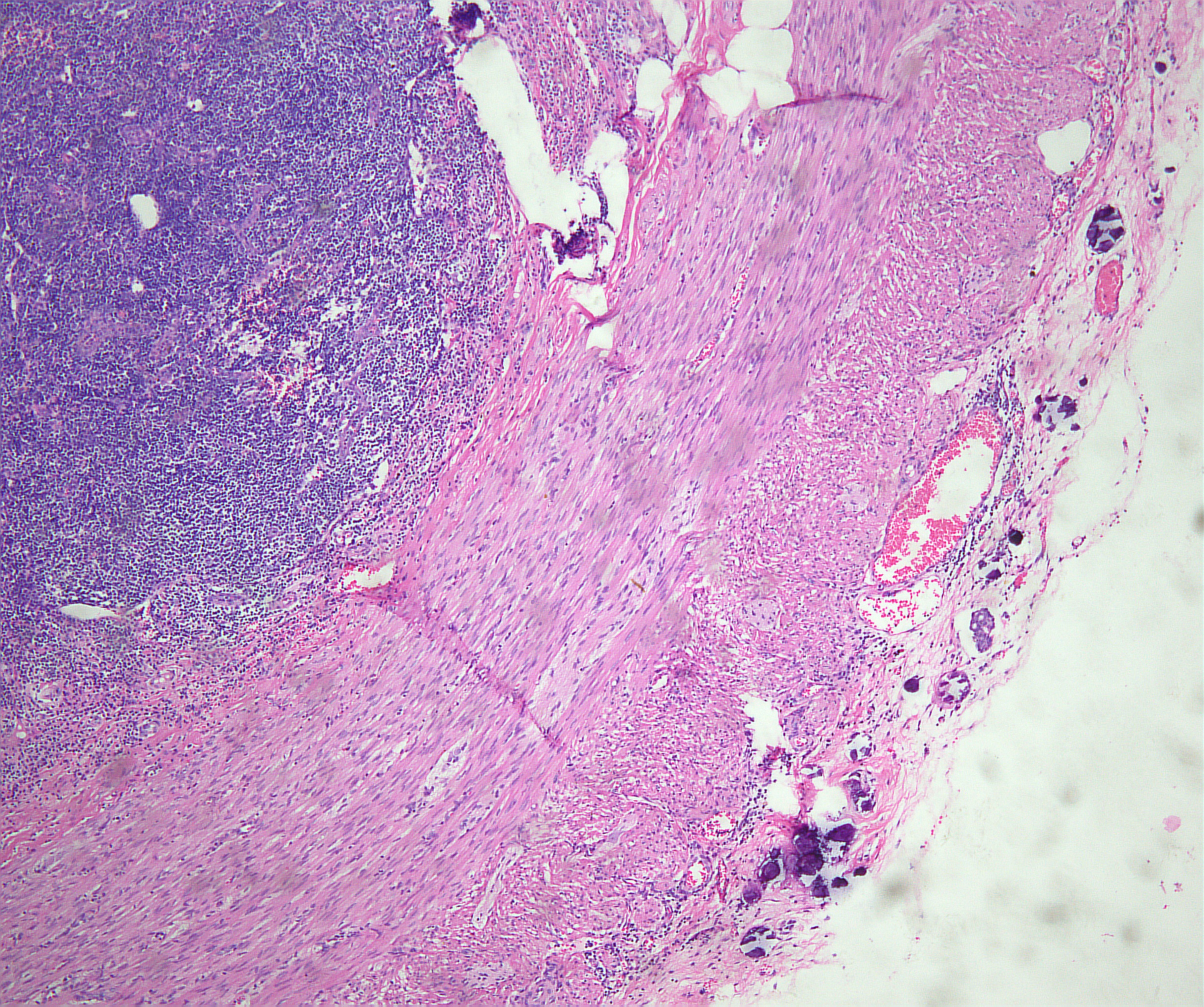

Supplement: Supplementary file 1 [file Image1.jpeg]

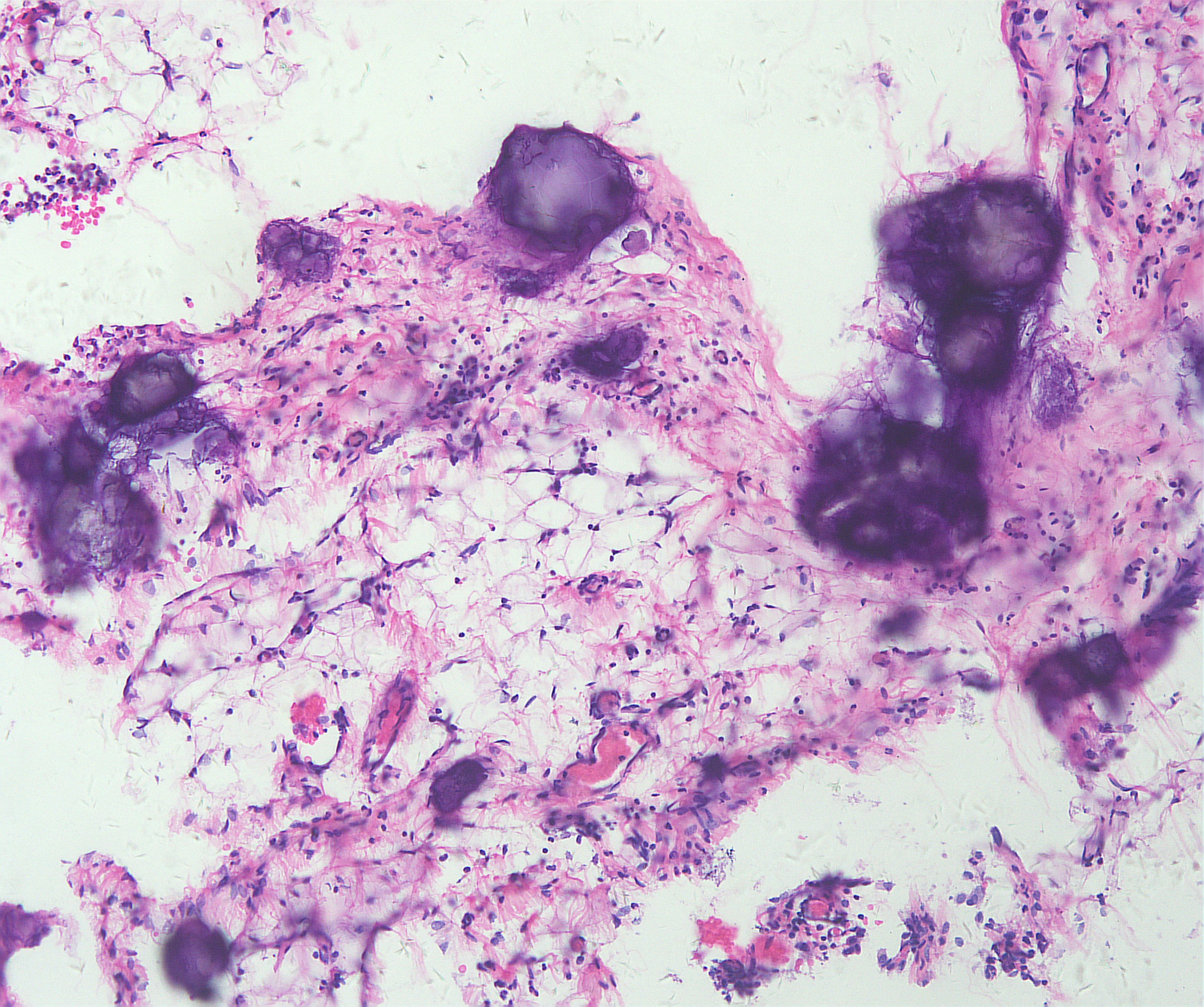

Supplement: Supplementary file 2 [file Image2.jpeg]
